# Internet addiction and mind-wandering in Chinese college students: chain mediation roles of physical activity and self-control

**DOI:** 10.3389/fpsyg.2026.1938850

**Published:** 2026-09-08

**Authors:** Wenjie Zhu, Wenping Zhu, Yizhuo Fan

**Affiliations:** 1 School of Physical Education, Guangzhou College of Commerce, Guangzhou, China; 2 School of Physical Education, Guangzhou Sport University, Guangzhou, China; 3 Guangdong University of Education, Guangzhou, China

**Keywords:** chain mediation model, college students, internet addiction, mind-wandering, physical activity, self-control

## Abstract

**Background:**

Against the backdrop of the deep integration of mobile internet technologies into university students’ daily lives, concerns regarding the potential impact of internet addiction on cognitive functioning have been increasing, while mind-wandering, as an important manifestation of impaired attentional control, remains insufficiently understood in terms of its underlying mechanisms.

**Objective:**

This study examined the relationship between internet addiction and mind-wandering among college students, along with the chain mediating roles of physical activity and self-control.

**Methods:**

A convenience sample of 3,472 Chinese college students completed the Internet Addiction Test—Short Form, the Mind-Wandering Questionnaire, the Physical Activity Rating Scale, and the Brief Self-Control Scale. Data analysis used SPSS 27.0 and AMOS 26.0 for descriptive, correlational, and structural equation modeling, with mediation effects tested via bootstrap (5,000 resamples).

**Results:**

Internet addiction showed a positive association with mind-wandering and negative associations with physical activity and self-control. Both physical activity and self-control showed negative associations with mind-wandering, while physical activity showed a positive association with self-control. Structural equation modeling indicated that internet addiction directly predicted mind-wandering and indirectly influenced it through the independent mediating effects of physical activity and self-control, as well as through a sequential chain mediation pathway.

**Conclusion:**

These findings point to both behavioral (physical activity) and cognitive (self-control) pathways through which internet addiction relates to mind-wandering. This integrated behavioral–cognitive framework informs understanding of the cognitive correlates of internet addiction and suggests directions for targeted interventions among college students.

## Introduction

Since the beginning of the 21st century, the rapid development of mobile internet technologies has triggered an unprecedented information revolution, with smartphones and tablets being deeply embedded in university students’ daily lives, making them a key population of internet users (Wang et al., 2025a). Although the convenience of internet technologies has greatly facilitated knowledge acquisition and interpersonal communication, excessive and uncontrolled internet use has raised growing concerns. Prolonged immersion in virtual environments has been shown to adversely affect cognitive functioning, including impairments in executive function (Aitken et al., 2025), attentional resource depletion (Fraiwan et al., 2026), and reduced self-monitoring ability (Jin et al., 2026). Beyond these cognitive deficits, research has increasingly focused on underlying vulnerability factors, such as early maladaptive schemas, pervasive cognitive and emotional patterns formed in childhood, which may predispose individuals to addictive behaviors (Sedighi et al., 2026). Recent studies have suggested that, with the normalization of internet use, internet addiction may exacerbate mind-wandering among college students, thereby increasing risks to academic adjustment and mental health. Against this backdrop, a meta-analysis reported that the prevalence of internet addiction among Chinese college students is 10.7%, which is significantly higher than that observed in the general adult population (Liu et al., 2021). Similarly, empirical evidence indicates a significant positive association between internet addiction and mind-wandering, suggesting that individuals with higher levels of addiction tend to exhibit more frequent mind-wandering (Li Y. et al., 2025).

Mind-wandering refers to a shift of attention away from the current task or external environment toward internally generated, task-unrelated thoughts (Smallwood and Schooler, 2006). It is important to note that mind-wandering is not inherently maladaptive; cognitive psychology research has demonstrated its adaptive functions, including future planning, creative problem-solving, and meaning-making (Baird et al., 2011; Mooneyham and Schooler, 2013). However, mind-wandering becomes problematic when it occurs frequently, spontaneously, and without meta-awareness, particularly in contexts requiring sustained attention and goal-directed performance (Smallwood and Schooler, 2015). Evidence suggests that frequent and uncontrollable mind-wandering is closely associated with negative outcomes, including reduced learning efficiency, impaired working memory (Kong et al., 2018), and lower subjective wellbeing (Smallwood and Schooler, 2015), and is therefore considered an important risk factor for cognitive performance and psychological adaptation. Importantly, deficits in executive functions, including impulse control, cognitive flexibility, and response inhibition, as well as difficulties in emotion regulation, have been identified as key mechanisms linking internet addiction to cognitive impairments (Fallah et al., 2025). Given its significant impact on learning and daily functioning, identifying the determinants of mind-wandering and exploring effective intervention pathways holds important theoretical and practical implications. The present study focuses specifically on the maladaptive aspect of mind-wandering, namely its occurrence as an unintended and task-irrelevant attentional shift that interferes with academic and daily functioning, rather than its deliberate or constructive forms. This focus is justified by the study context: the participants are college students embedded in academic environments that demand sustained attention, and the predictor variable, internet addiction, is conceptualized as a risk factor that impairs cognitive control, making the pathological dimension of mind-wandering particularly relevant. Previous studies have indicated that internet addiction not only directly predicts mind-wandering among college students but may also indirectly influence it through factors such as psychological resilience, academic burnout, and anxiety (Qiu et al., 2025).

However, existing research has primarily focused on emotional or psychological adaptation pathways, while evidence from behavioral and cognitive regulation perspectives remains limited. Physical activity and self-control represent important behavioral and cognitive resources, both of which are closely related to internet addiction and mind-wandering; however, their sequential roles in explaining this relationship have not been fully examined. Therefore, this study develops a chain mediation model involving physical activity and self-control to elucidate the underlying mechanism through which internet addiction influences mind-wandering among college students, and to provide theoretical evidence for interventions targeting internet addiction and the improvement of cognitive functioning.

### The mediating role of physical activity

Physical activity refers to any bodily movement produced by skeletal muscles that results in energy expenditure. It has been identified as a key protective factor against mind-wandering (Morava et al., 2025). Attention control theory suggests that regular physical activity enhances prefrontal cortical activation and improves neurotransmitter efficiency, thereby optimizing the allocation and maintenance of attentional resources (McMorris, 2016). Empirical evidence from cross-sectional studies indicates a significant negative association between physical activity levels and mind-wandering among college students (Deng et al., 2022). Experimental findings further demonstrate that sustained engagement in physical activity strengthens functional connectivity within the executive control network while suppressing overactivation of the default mode network, thereby may reducing the occurrence of mind-wandering (Cline et al., 2024). However, this relationship has not been consistently supported across all studies; some research has also found that extremely high-intensity exercise may induce central fatigue and transiently impair attentional control, paradoxically increasing mind-wandering (Chang et al., 2014). These inconsistent findings suggest that the relationship between physical activity and mind-wandering warrants further empirical examination.

However, internet addiction has been shown to substantially reduce individuals’ participation in physical activity. The time displacement hypothesis posits that excessive internet use displaces time originally allocated to physical activity and undermines motivation for real-world engagement (Wang et al., 2025b). Both cross-sectional and longitudinal evidence suggest that internet addiction is a significant risk factor for reduced physical activity (Wang et al., 2024c), and prospective studies further confirm that higher levels of internet addiction are associated with lower physical activity over time (Fan et al., 2024). However, a few studies have observed a weak positive association in specific subgroups, particularly when internet use is oriented toward exercise-related content (Liu et al., 2022). Thus, the relationship between internet addiction and physical activity remains controversial in the existing literature and awaits further clarification through additional research.

### The mediating role of self-control

In addition to reduced engagement in physical activity, impaired self-control has been identified as another critical cognitive pathway through which internet addiction may increase mind-wandering (Li J. et al., 2025), particularly among college students who are in a key developmental stage of cognitive control and personality maturation. According to the self-control strength model (Baumeister et al., 1998), self-control is a limited psychological resource that can be depleted through sustained exertion, ultimately leading to self-regulatory failure. As a form of frequent impulsive behavior, internet addiction continuously consumes self-control resources, thereby reducing individuals’ capacity to maintain attentional monitoring and inhibit irrelevant distractions in subsequent cognitive tasks (Guo et al., 2022). In addition, the immediate gratification associated with addictive internet use may weaken individuals’ preference for delayed rewards, further undermining their ability to resist internal distractions and maintain goal-directed behavior (Acuff et al., 2022). Cross-sectional evidence also indicates that college students with higher levels of internet addiction tend to report significantly lower self-control scores compared with their peers, and such deficits in cognitive regulation are associated with a greater likelihood of task-unrelated thought (Liu, 2014).

Conversely, self-control is considered a key determinant in suppressing mind-wandering and sustaining stable attentional focus. Individuals with higher self-control ability are better able to maintain goal-directed attention, inhibit spontaneous activation of the default mode network, and reduce interference from task-unrelated internal thoughts (Andrews-Hanna et al., 2014). Moreover, self-control enhances top-down regulation from the prefrontal cortex over subcortical structures, such as the posterior cingulate cortex and medial prefrontal cortex, thereby attenuating the neural basis of mind-wandering (Golchert et al., 2017) and supporting sustained allocation of attentional resources to external tasks (Stawarczyk et al., 2021). Based on these theoretical and empirical findings, it is proposed that internet addiction may impair self-control resources among college students, which in turn increases their level of mind-wandering.

### The chain mediation role of physical activity and self-control

This study aims to examine the mediating roles of physical activity and self-control in the relationship between internet addiction and mind-wandering among Chinese college students, with the goal of advancing understanding of the underlying mechanisms through which internet addiction influences mind-wandering. As discussed above, both physical activity and self-control may help explain the increased tendency toward mind-wandering among college students. It is therefore proposed that these two factors may operate in a sequential chain mediation process; however, the exact ordering and interplay between them remain to be empirically tested. Clarifying this relationship is essential for revealing the multi-layered and multi-pathway mechanisms linking internet addiction to mind-wandering.

Existing theories provide a basis for understanding the association between physical activity and self-control, while also suggesting that internet addiction may indirectly weaken self-control by disrupting this relationship. The self-control strength model posits that self-control is a limited psychological resource that can be strengthened through repeated practice, and regular physical activity itself can be viewed as a form of self-control training (Fan and Li, 2025). Intervention studies have provided direct evidence for this view, demonstrating that regular physical activity training can effectively enhance individuals’ self-control ability (Xu et al., 2025). From a neurobiological perspective, regular physical activity can enhance the structure and function of the prefrontal cortex, which is precisely the core brain region supporting self-control (Hillman et al., 2008), thereby providing a neural foundation for the relationship between physical activity and improved self-control. Self-determination theory further suggests that physical activity satisfies basic psychological needs for autonomy, competence, and relatedness, thereby fostering autonomous motivation that facilitates more effective self-regulation (Ryan and Deci, 2017; Vaquero Solís et al., 2019). Conservation of resources theory also indicates that the physical and psychological resource gains derived from physical activity can enhance cognitive control and behavioral inhibition capacities (Castonguay et al., 2018). Taken together, regular physical activity should contribute to the development and strengthening of self-control; however, internet addiction significantly reduces engagement in physical activity among college students, thereby disrupting this positive developmental pathway. As a result, individuals lose opportunities to enhance self-control through physical activity, leading to progressive depletion of self-control resources and an increased risk of mind-wandering. In summary, the negative effect of internet addiction on mind-wandering may be explained by a dual pathway involving reduced physical activity and weakened self-control.

### The present study

**FIGURE 1:**
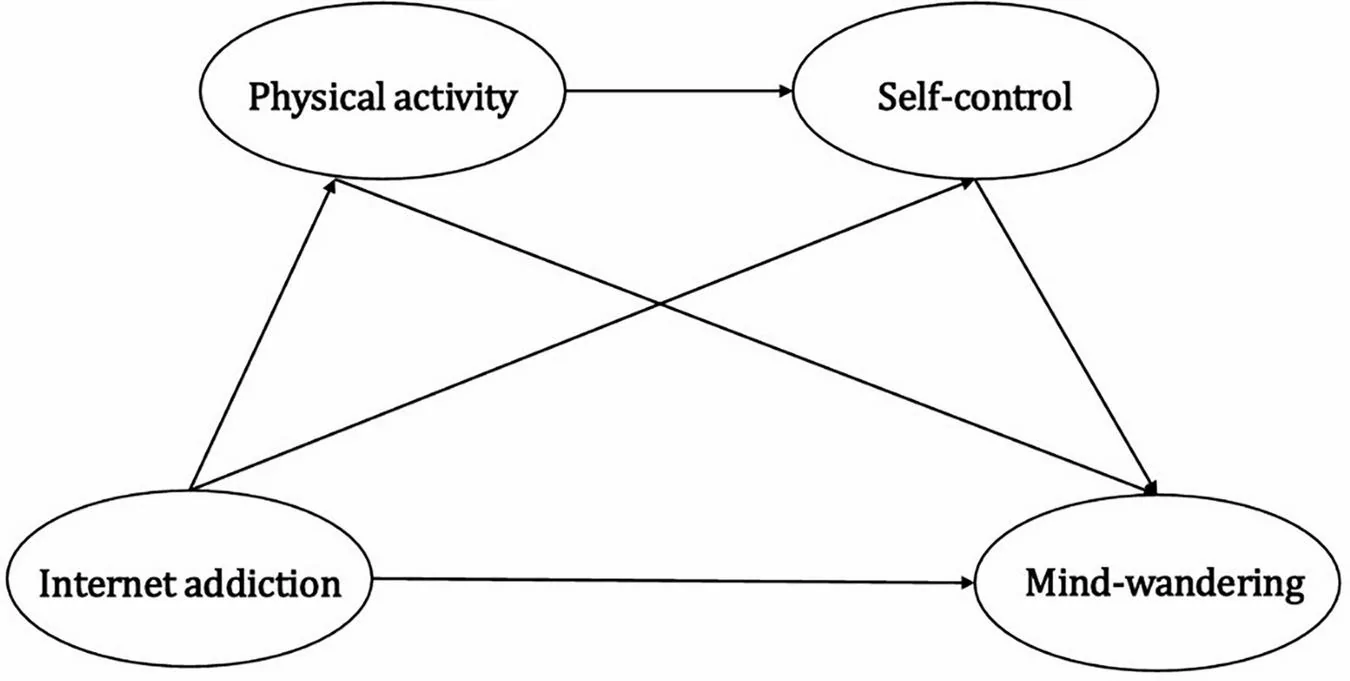
The hypothesized sequential mediation model of the present study.

Building on the preceding analyses, internet addiction, physical activity, self-control, and mind-wandering are interrelated in a complex system of associations. Although prior research has predominantly examined bivariate relationships among these constructs in isolation, the full sequential process through which internet addiction influences mind-wandering via physical activity and self-control remains insufficiently understood. The novelty of this study is reflected in two aspects. First, mind-wandering, as a core cognitive indicator of attentional control impairment, has not received sufficient attention in internet addiction research, with most existing studies focusing on outcome variables such as academic performance or general mental health. Second, although previous studies have separately examined the mediating roles of self-control or physical activity, no study has incorporated both into a unified sequential framework for examination. To address the above research gaps, the present study integrates the time displacement hypothesis, the strength model of self-control, and attentional control theory to construct a chain mediation model of physical activity → self-control, proposing a “behavior–cognition” interaction pathway through which internet addiction is associated with increased mind-wandering via reduced physical activity participation and depleted self-control resources. It should be noted that the order of variables in the model was theoretically derived based on existing theories and prior empirical evidence, rather than being established through empirically tested temporal sequencing. Based on the theoretical framework and proposed mechanisms, the following hypotheses are advanced (see Figure 1):

**Hypothesis 1:** Internet addiction is positively associated with mind-wandering among college students.

**Hypothesis 2:** Physical activity plays a mediating role in the relationship between internet addiction and mind-wandering.

**Hypothesis 3:** Self-control plays a mediating role in the relationship between internet addiction and mind-wandering.

**Hypothesis 4:** Physical activity and self-control serve as sequential mediators in the relationship between internet addiction and mind-wandering.

## Materials and methods

### Participants and procedure

Prior to formal data collection*, a priori* sample size estimation was conducted to ensure the stability of structural equation modeling (SEM) analyses. Based on relevant recommendations, the minimum required sample size should be at least ten times the number of observed variables (Lu et al., 2026). Given that the present study included 27 observed variables, the minimum required sample size was estimated to be 270. Accordingly, the target sample size was set at above 270, with an additional 20% contingency margin to account for invalid responses and potential misunderstandings, resulting in a planned distribution of no fewer than 324 questionnaires.

Data were collected using a convenience sampling strategy between January and March 2026. Participants were recruited through online platforms, including advertisements posted on WeChat public accounts, Weibo, and Xiaohongshu, as well as targeted invitations distributed through online student communities. Inclusion criteria required participants to be currently enrolled in universities in mainland China and capable of independently completing an online questionnaire. To identify duplicate responses, the questionnaire platform was set to allow each WeChat account to complete the survey only once, and IP addresses were also checked; no duplicate submissions were found. To verify participant eligibility, the questionnaire required respondents to indicate the name of their institution and confirm their current enrollment status at the beginning of the survey. A total of 3,816 questionnaires were distributed. After data screening, 344 questionnaires were excluded, yielding 3,472 valid responses (effective response rate = 90.98%). Among the excluded questionnaires, 218 were removed due to missing data (more than 20% of items unanswered), 76 due to patterned responses (e.g., selecting the same option for all items, with a standard deviation of scale items ≤ 0.5), and 50 due to logically inconsistent responses (e.g., contradictory answers to reverse-scored items). Missing data occurred at the item level rather than the questionnaire level. Because questionnaires with more than 20% missing items were excluded prior to analysis and no imputation was performed for remaining missing values, complete-case analysis was adopted, and all analyses were based on the 3,472 participants with complete responses. The mean age of participants was 18.78 years (SD = 0.94). Females accounted for 58.5% of the sample, while males accounted for 41.5%. Detailed demographic characteristics are presented in Table 1. This study was conducted in accordance with the Declaration of Helsinki and was approved by the Research Ethics Committee of Guangzhou College of Commerce (approval no. CGCC-SPT-2025-012). All participants provided electronic informed consent prior to participation and were informed of their right to withdraw at any time.

**TABLE 1:** Demographic characteristics of the participants.

Variable	Category	Frequency	Percentage (%)
Gender	Male	1,442	41.5
	Female	2,030	58.5
Grade	Freshman	2,026	58.4
	Sophomore	829	23.9
	Junior	561	16.2
	Senior	48	1.4
	Graduate student	8	0.2
Residential area	Urban	1,016	29.3
	Suburban	771	22.2
	Rural	1,685	48.5
Self-rated health status	Very good	494	14.2
	Good	1,477	42.5
	Fair	1,310	37.7
	Unhealthy	153	4.4
	Very unhealthy	38	1.1

### Measures

#### Internet addiction

Internet addiction was measured using the Chinese version of the Internet Addiction Test—Short Form (IAT-7). This scale is a seven-item brief version developed by Valenti et al. (2023) based on Young’s (1998) Internet Addiction Test, comprising two factors: interpersonal, emotional, and compulsive conflict (five items) and online time management (two items). A sample item is “Do you find that you stay online longer than you intended?” Responses were rated on a five-point Likert scale ranging from 1 (“never”) to 5 (“always”), with higher scores indicating stronger tendencies toward internet addiction. As no published Chinese validation study is currently available for this scale, the present study employed a standard translation procedure to translate it into Chinese: two bilingual translators independently conducted forward translations, a third translator performed blind back-translation and compared it with the original, the research team reached a consensus through discussion, and a pilot test was conducted in a small sample of college students to examine item comprehensibility. In the present sample, confirmatory factor analysis further demonstrated an acceptable model fit (TLI = 0.909, CFI = 0.939, SRMR = 0.042, RMSEA = 0.074), supporting the factorial validity of the scale. Reliability analyses showed good internal consistency (Cronbach’s α = 0.881; Guttman split-half coefficient = 0.833), indicating that the scale reliably captures individuals’ levels of internet addiction.

#### Mind-wandering

Mind-wandering was assessed using the Chinese version of the Mind-Wandering Questionnaire (Mrazek et al., 2013), which was translated and validated in Chinese college students (引用：心理游离问卷中文版的修订及其信效度研究). The scale consists of five items rated on a six-point Likert scale ranging from 1 (“almost never”) to 6 (“almost always”). The total score was computed by summing all items, with higher scores indicating a stronger tendency toward mind-wandering. Confirmatory factor analysis demonstrated excellent model fit (TLI = 0.991, CFI = 0.993, SRMR = 0.009, RMSEA = 0.057), supporting the construct validity of the scale. Reliability analyses further indicated strong internal consistency (Cronbach’s α = 0.927; Guttman split-half coefficient = 0.885), confirming that the instrument reliably measures participants’ mind-wandering tendencies.

#### Physical activity

Physical activity was assessed using the Physical Activity Rating Scale (PARS-3) developed by Liang (1994). The scale evaluates individuals’ physical activity levels across three dimensions: exercise intensity, frequency, and duration, and has been widely applied in Chinese sports science research. Physical activity volume was calculated using the formula: exercise volume = intensity × (duration−1) × frequency, with higher scores indicating greater levels of physical activity participation. It should be noted that the PARS-3 is a well-established index-based measurement tool rather than a reflective latent construct. Its score is derived from a multiplicative algorithm combining three indicators, and the scale contains only three items; therefore, it does not meet the methodological requirements for confirmatory factor analysis. Its reliability and validity have been repeatedly verified in previous studies among Chinese college students (Wang et al., 2025a), and the present study followed the standardized administration and scoring procedures. In the current sample, the scale demonstrated acceptable internal consistency (Cronbach’s α = 0.656). It is important to note that although 0.656 falls below the conventional threshold of 0.70, the multiplicative scoring structure inherently weakens the linear relationships among items, thereby yielding a relatively low Cronbach’s α value (Streiner et al., 2003). In the field of physical activity epidemiology, α ≥ 0.6 is generally considered an acceptable level of reliability, particularly in exploratory research (Chen and Zhang, 2026).

#### Self-control

Self-control was measured using the revised Chinese version of the Brief Self-Control Scale (Luo et al., 2021), which consists of seven items. Responses were rated on a five-point Likert scale ranging from 1 (“never”) to 5 (“always”). The scale includes both positively and negatively worded items, and reverse scoring was applied to negatively worded items prior to data analysis. Higher total scores indicate stronger self-control ability. Confirmatory factor analysis further demonstrated excellent model fit (TLI = 0.994, CFI = 0.996, SRMR = 0.009, RMSEA = 0.011), supporting the structural validity of the scale. Reliability analysis showed acceptable internal consistency (Cronbach’s α = 0.776; Guttman split-half coefficient = 0.778), indicating that the scale reliably captures participants’ self-control levels.

#### Demographic variables

In addition to the primary study variables, basic demographic information was collected, including age (years), gender (1 = male, 2 = female), academic year (1 = first year, 2 = second year, 3 = third year, 4 = fourth year, 5 = postgraduate), residential background (1 = urban, 2 = suburban, 3 = rural), and self-rated health status (1 = very unhealthy, 2 = unhealthy, 3 = average, 4 = healthy, 5 = very healthy). All of these variables were included as control variables in subsequent analyses to account for potential confounding effects.

### Statistical analysis

Data analyses were conducted using SPSS 27.0 and AMOS 26.0. First, descriptive statistics were computed to examine the mean values, standard deviations, and other basic characteristics of the variables, thereby providing an overview of the sample distribution. Harman’s single-factor test was applied to assess potential common method bias. Normality tests were performed for all core variables. Physical activity showed a degree of non-normality and was therefore transformed prior to correlation analysis. In addition, the absolute values of skewness and kurtosis for internet addiction, mind-wandering, and self-control were all less than 2, indicating approximate normal distributions. Pearson correlation analyses were then conducted to examine the associations among internet addiction, physical activity, self-control, and mind-wandering. For hypothesis testing, a structural equation modeling approach was employed to construct a chain mediation model, in which internet addiction served as the independent variable, mind-wandering as the dependent variable, and physical activity and self-control as sequential mediators. Relevant demographic variables were included in the model as covariates. Model parameters were estimated using the maximum likelihood method, and the significance of path coefficients was evaluated using bias-corrected bootstrap procedures with 5,000 resamples. This bootstrap method does not require the assumption of multivariate normality and therefore provides robust standard errors and confidence intervals for the indirect effects. Indirect effects were considered significant when the 95% confidence intervals did not include zero. Model fit was evaluated using fit indices that are less sensitive to sample size, including the comparative fit index (CFI), Tucker–Lewis index (TLI), standardized root means square residual (SRMR), and root mean square error of approximation (RMSEA). Given that the chi-square statistic is highly sensitive to large sample sizes and may over-reject well-fitting models (Chen, 2007), it was not used as a primary criterion for model evaluation. Model fit was considered acceptable when CFI and TLI were ≥0.90 and both RMSEA and SRMR were <0.08 (Wang et al., 2025c). All analyses were conducted using two-tailed tests, with a significance level set at α = 0.05.

## Results

### Common method bias analysis

Given that all variables in the present study were measured using self-reported data, potential common method bias may exist. To address this concern, Harman’s single-factor test was conducted. The results of the unrotated exploratory factor analysis indicated that multiple factors with eigenvalues greater than 1 were extracted, and the first factor accounted for 28.91% of the total variance, which is below the commonly accepted threshold of 40% (Wang et al., 2024b). These findings suggest that common method bias does not pose a serious threat to the validity of the present study. However, Harman’s single factor test is considered a comparatively weak approach for assessing common method variance (Podsakoff et al., 2003). To address this limitation, we further employed the unmeasured latent variable technique as a supplementary test. Comparing models with and without a latent method factor revealed negligible improvement in fit indices (ΔCFI = 0.002, ΔTLI = 0.003, ΔRMSEA = 0.003, ΔSRMR = 0.004) (Tang and Wen, 2020). This result indicates that common method bias does not pose a major threat to the findings.

### Participant characteristics

A total of 3,472 valid responses from college students were included in the final analysis. The demographic characteristics of the sample are as follows. In terms of gender distribution, 1,442 participants were male (41.5%) and 2,030 were female (58.5%), indicating a slightly higher proportion of female respondents. Regarding academic year, 2,026 participants were in their first year (58.4%), 829 in their second year (23.9%), 561 in their third year (16.2%), 48 in their fourth year (1.4%), and eight were postgraduate students (0.2%), indicating that the sample was primarily composed of lower-year undergraduates, with first-year students accounting for nearly 60%. With respect to residential background, the largest proportion of participants came from rural areas (*n* = 1,685, 48.5%), followed by urban areas (*n* = 1,016, 29.3%) and suburban areas (*n* = 771, 22.2%), indicating a relatively balanced representation across different residential contexts. Regarding self-rated health status, most participants reported relatively positive health conditions, with 1,477 (42.5%) rating themselves as “healthy” and 494 (14.2%) as “very healthy,” accounting for 56.7% in total. A total of 1,310 participants (37.7%) reported their health as “average,” while only 191 (5.5%) reported being “unhealthy” or “very unhealthy.” Overall, the sample exhibited a moderately favorable self-rated health profile. Detailed frequency distributions of all variables are presented in Table 1.

### Descriptive statistics and correlation analysis

Due to the physical activity variable did not conform to a normal distribution, a normality transformation was conducted prior to correlation analysis. Pearson’s product–moment correlation analysis was then used to examine the relationships among the study variables. As shown in Table 2, internet addiction was significantly positively associated with mind-wandering (*r* = 0.610, *p* < 0.001), and significantly negatively associated with physical activity (*r* = −0.229, *p* < 0.01) and self-control (*r* = −0.436, *p* < 0.01). Mind-wandering was also significantly negatively correlated with physical activity (*r* = −0.254, *p* < 0.01) and self-control (*r* = −0.479, *p* < 0.01). In addition, physical activity was positively correlated with self-control (*r* = 0.355, *p* < 0.01).

**TABLE 2:** Correlation matrix among study variables (*N* = 3,472).

Variables	*M*	SD	1	2	3	4
1. Mind-wandering	13.34	3.786	1	–	–	–
2. Physical activity	2.586	2.111	−0.254**	1	–	–
3. Self-control	21.77	5.198	−0.479**	0.355**	1	–
4. Internet addiction	19.99	5.099	0.610***	−0.229*	−0.436**	1

M, mean score; SD, standard deviation. *p < 0.05; **p < 0.01; ***p < 0.001.

To further examine potential multicollinearity among variables, regression analyses were conducted with internet addiction, physical activity, and self-control as predictors. The results indicated that variance inflation factor (VIF) values ranged from 1.153 to 1.350, and tolerance values ranged from 0.741 to 0.867, all within acceptable thresholds. These findings suggest that no multicollinearity issues were present, supporting the suitability of the data for subsequent path analysis.

### Testing for chain mediation effects

#### Model fit

Based on the prerequisite that the reliability and validity of the measurement instruments were adequately established, the present study further controlled for relevant covariates and examined the fit between the structural equation model and the observed data (see Table 3). The results indicated that both the CFI and TLI exceeded the recommended threshold of 0.90, while the root mean RMSEA and SRMR were below the acceptable criterion of 0.08. Taken together, these fit indices suggest that the proposed theoretical model demonstrates satisfactory structural validity and meets the statistical requirements for subsequent path analysis.

**TABLE 3:** Goodness-of-fit indices for the structural model.

Category	Fit index	Fit indices	Recommended threshold
Absolute fit indices	SRMR	0.031	<0.08
	RMSEA	0.060	<0.08
Incremental fit indices	TLI	0.955	>0.90
	CFI	0.964	>0.90

CFI, comparative fit index; TLI, Tucker–Lewis index; RMSEA, root mean square error of approximation; SRMR, standardized root mean square residual.

#### Direct effects

**FIGURE 2:**
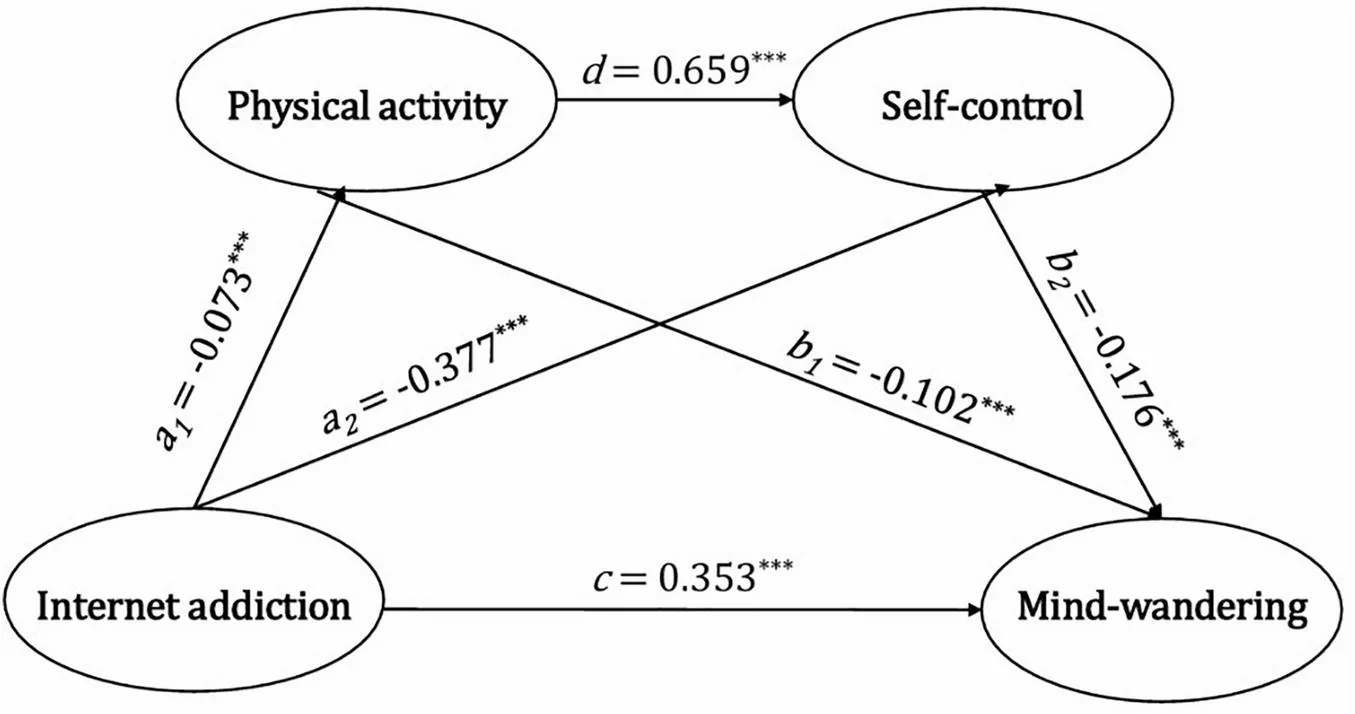
Final model of the present study. a_1_, a_2_, b_1_, b_2_, c, d, unstandardized coefficient; ***p < 0.001.

On the basis of an acceptable model fit, the structural equation model was estimated using AMOS with the maximum likelihood method (see Table 4). Standardized path coefficients were used to indicate the direction and strength of the relationships among variables, and the specific structural paths are illustrated in Figure 2. After controlling for demographic covariates, regression results indicated that internet addiction showed significant negative associations with physical activity (B = −0.073, *p* < 0.001) and self-control (B = −0.377, *p* < 0.001), and a significant positive association with mind-wandering (B = 0.353, *p* < 0.001). In addition, physical activity showed a significant positive effect on self-control (B = 0.659, *p* < 0.001) but a significant negative effect on mind-wandering (B = −0.102, *p* < 0.001). Self-control was also significantly negatively associated with mind-wandering (B = −0.176, *p* < 0.001). Regarding explanatory power, the model accounted for 12.0% of the variance in physical activity (R^2^ = 0.120), 25.9% of the variance in self-control (R^2^ = 0.259), and 44.1% of the variance in mind-wandering (R^2^ = 0.441). The corresponding F-statistics were all statistically significant (*F* = 78.79, 173.6, and 341.3, respectively, *p* < 0.001), indicating satisfactory overall model fit.

**TABLE 4:** Testing the interconnections among variables.

Variables	Physical activity			Self-control			Mind-wandering		
	B	SE	*t*	B	SE	*t*	B	SE	*t*
Constant	6.262***	0.857	7.304	28.80***	1.951	14.76	9.354	1.273	7.343
Age	0.033	0.047	0.703	−0.054	0.106	−0.514	0.059	0.067	0.880
Gender	−0.754***	0.071	−10.62	0.090	0.162	0.553	−0.550	0.103	−5.333
Grade	−0.130*	0.056	−2.311	0.091	0.127	0.713	−0.068	0.080	−0.851
Residential area	−0.185***	0.039	−4.680	−0.073	0.089	−0.815	0.076	0.056	1.347
Self-rated health status	−0.431***	0.042	−10.10	−0.120	0.097	−1.238	0.313	0.061	5.068
Internet addiction	−0.073***	0.006	−10.77	−0.377***	0.015	−24.07	0.353***	0.011	32.95
Physical activity	–	–	–	0.659***	0.038	17.18	−0.102***	0.025	−4.038
Self-control	–	–	–	–	–	–	−0.176***	0.011	−16.44
R	0.346	–	–	0.509	–	–	0.664	–	–
R^2^	0.120	–	–	0.259	–	–	0.441	–	–
*F*	78.79***	–	–	173.6***	–	–	341.3***	–	–

B, unstandardized coefficient; SE, standard error. *p < 0.05; ***p < 0.001.

Taken together, the results of the path analysis suggest that internet addiction not only exerts a direct effect on mind-wandering but also operates through multiple indirect pathways: (1) via reduced physical activity, (2) via impaired self-control, and (3) via a sequential mediation pathway in which internet addiction first reduces physical activity, which in turn weakens self-control, ultimately leading to increased mind-wandering. These findings provide empirical support for the proposed chain mediation hypothesis.

#### Mediation effects

To further examine the mediating roles of physical activity and self-control in the relationship between internet addiction and mind-wandering, a bootstrap analysis was conducted with 5,000 resamples to estimate indirect effects and their 95% confidence intervals (see Table 5). The results indicated that all three indirect pathways were statistically significant, as their confidence intervals did not include zero. Specifically, the indirect effect of internet addiction on mind-wandering via physical activity was 0.007 (95% CI [0.003, 0.012]), accounting for 1.61% of the total effect. The indirect effect via self-control was 0.068 (95% CI [0.057, 0.076]), accounting for 15.60% of the total effect. The sequential mediation pathway through physical activity and self-control was 0.008 (95% CI [0.006, 0.011]), accounting for 1.83% of the total effect. The total indirect effect was 0.083, representing 19.04% of the total effect. After controlling for mediators, the direct effect of internet addiction on mind-wandering remained statistically significant (β = 0.353, 95% CI [0.332, 0.374]), accounting for 80.96% of the total effect. The total effect was 0.436 (95% CI [0.416, 0.456]). These indirect effects should be interpreted as statistical associations observed in cross-sectional data, consistent with the theoretically proposed mediation model, rather than as evidence of a verified temporal or causal sequence. Overall, these findings suggest that self-control contributes more substantially than physical activity to the mediating process and represents a key mechanism linking internet addiction to mind-wandering. Meanwhile, physical activity participates in this relationship both directly and indirectly through the sequential pathway, supporting a coordinated interaction between behavioral and cognitive mechanisms in explaining mind-wandering.

**TABLE 5:** Testing for chain mediation effects.

Paths	Effect	Boot SE	Bootstrap 95% CI	Effect size ratio
Internet addiction → physical activity →mind-wandering	0.007	0.002	[0.003, 0.012]	1.61%
Internet addiction → self-control →mind-wandering	0.068	0.004	[0.057, 0.076]	15.60%
Internet addiction → physical activity → self-control →mind-wandering	0.008	0.002	[0.006, 0.011]	1.83%
Total indirect effect between internet addiction and mind-wandering	0.083	0.005	[0.072, 0.093]	19.04%
Direct effect	0.353	0.011	[0.332, 0.374]	80.96%
Total effect	0.436	0.010	[0.416, 0.456]	100%

CI, confidence interval; SE, standard error.

## Discussion

This study investigated the mechanisms underlying the relationship between internet addiction and mind-wandering among college students, with a particular focus on the mediating roles of physical activity and self-control. Given the cross-sectional nature of the data, the reported mediation pathways should be interpreted as statistical associations consistent with the theoretical model rather than as causal inferences, and all directional interpretations are based on theoretical assumptions rather than empirical evidence of temporal sequencing. The results showed that internet addiction directly predicted mind-wandering and also showed indirect effects through independent mediating pathways via physical activity and self-control, as well as through a sequential chain mediation pathway involving both variables. These findings offered empirical support for the proposed hypotheses and added new evidence for understanding the mechanisms in question.

### Internet addiction and mind-wandering

Consistent with previous research (Li J. et al., 2025), the present study found a significant positive association between internet addiction and mind-wandering among college students. Specifically, higher levels of internet addiction were associated with more frequent occurrences of mind-wandering, further confirming the relationship between internet addiction and attentional control. This study suggests several possible mechanisms underlying this association. First, from a cognitive resource perspective, internet addiction may continuously consume executive control resources, thereby weakening individuals’ ability to maintain goal-directed attention and inhibit irrelevant information (McVay and Kane, 2009). When executive control is compromised, individuals are more likely to disengage from ongoing tasks and shift toward internally generated thoughts, increasing the likelihood of mind-wandering. Second, internet addiction often co-occurs with reduced physical activity (Wang et al., 2025b), poorer sleep quality (Bai et al., 2024), and other unhealthy lifestyle behaviors, all of which have been linked to attentional control and abnormal activation of the default mode network. In addition, internet addiction frequently coincides with psychological problems such as anxiety (Qiu et al., 2025) and negative affect (Wang et al., 2023), which may disrupt attentional resource allocation and increase task-unrelated thinking. Overall, these findings suggest that internet addiction is not only a behavioral issue but also a notable factor related to cognitive functioning and attentional stability among college students.

### The mediating role of physical activity

The results supported Hypothesis 2, confirming that physical activity plays a significant mediating role in the relationship between internet addiction and mind-wandering. This finding extends existing research on the interplay among internet addiction, physical activity, and mind-wandering, and provides preliminary support for the intersection of the time displacement hypothesis and neuroplasticity theory. Specifically, the time displacement hypothesis proposes that internet use, as a highly engaging alternative activity, displaces time that would otherwise be allocated to physical activity (Wang et al., 2025b). When college students devote substantial leisure time to virtual environments, their opportunities for engaging in physical activity are substantially reduced, which in turn limits the physiological foundation necessary for optimal cognitive functioning. Neuroplasticity theory further indicates that physical activity-induced neural adaptations are associated with enhanced prefrontal cortical activation, greater stability of executive control networks, and suppressed overactivation of the default mode network (Buckley et al., 2014). Accordingly, higher levels of internet addiction are associated with lower physical activity participation, and this pattern is in turn associated with higher frequency of mind-wandering. This finding also suggests that promoting physical activity may benefit not only physical health but also serve as a potential intervention strategy for addressing the cognitive correlates of internet addiction.

### The mediating role of self-control

In addition, the results demonstrated that self-control significantly mediated the relationship between internet addiction and mind-wandering, thereby supporting Hypothesis 3. This finding points to the depletion of self-control resources as an important psychological mechanism in the link between internet addiction and mind-wandering. According to the self-control strength model (Baumeister et al., 1998), self-control is a limited psychological resource that must be continuously allocated to regulate behavior and attention. Individuals with internet addiction are frequently exposed to immediate rewards stimuli, which fosters a preference for instant gratification and is associated with continuous depletion of self-control resources. When self-control resources are insufficient, individuals’ ability to suppress irrelevant thoughts and maintain task goals is weakened (Andrews-Hanna et al., 2014), making them more susceptible to attentional drift and mind-wandering. Moreover, self-control is regarded as a key protective factor for maintaining cognitive stability and goal-directed behavior. Higher levels of self-control are associated with enhanced ability to regulate attentional resources and improved efficiency in inhibiting internal distractions, which in turn is associated with a lower likelihood of mind-wandering (Saxena, 2024). Thus, depletion of self-control resources represents a critical pathway through which internet addiction is related to cognitive functioning among college students. This is consistent with findings that interventions targeting executive functions and emotion regulation, such as body awareness psychotherapy, can effectively reduce internet addiction by improving impulse control, cognitive flexibility, and response inhibition (Fallah et al., 2025). It is worth noting that the independent mediating effect of physical activity (1.61%) was considerably smaller than that of self-control (15.60%). This disparity can be understood in terms of the underlying mechanisms: self-control directly reflects the depletion of attention maintenance and impulse inhibition capabilities resulting from internet addiction, and thus constitutes the dominant pathway through which internet addiction affects mind-wandering. In contrast, physical activity exerts its influence on attentional control indirectly through long-term physiological adaptations and behavioral changes, and its effect is therefore relatively limited. Although these indirect effects were statistically significant, the relatively small magnitude of the physical activity-related effects in the context of a large sample underscores the distinction between statistical significance and practical importance. This suggests that physical activity plays a supplementary rather than a dominant role in the relationship between internet addiction and mind-wandering, and its practical implications should be interpreted with caution.

It is also important to consider possible alternative directions of association and reciprocal causality. For example, although our theoretical model assumes that internet addiction impairs self-control, the reverse pathway is equally plausible: individuals with lower baseline self-control may be more susceptible to developing internet addiction (Zhao et al., 2024). Likewise, mind-wandering may increase susceptibility to internet addiction by reducing awareness of excessive use, rather than being merely a consequence of internet addiction (Müller et al., 2021). Furthermore, a bidirectional relationship among these variables is also possible, as diminished self-control may reinforce compulsive internet use, which in turn further depletes self-control resources. Given that the cross-sectional design of this study cannot distinguish among these competing explanations, these alternative pathways await investigation in future longitudinal or experimental studies.

### The chain mediation role of physical activity and self-control

Finally, the results further supported Hypothesis 4, indicating that internet addiction is associated with mind-wandering through a sequential mediation pathway involving physical activity and self-control. Specifically, higher levels of internet addiction were associated with lower engagement in physical activity, which in turn was associated with lower self-control capacity, and this pattern was further associated with higher levels of mind-wandering. Existing research has suggested that physical activity is not only a form of physical exercise but also a continuous process of self-regulation training. Regular participation in physical activity requires individuals to overcome inertia, persist toward goals, and regulate behavior, and these processes are related to the enhancement of self-control. From a neurobiological perspective, physical activity is associated with the development of prefrontal cortical functioning and improved executive control efficiency, providing a neural foundation for self-control (Buckley et al., 2014). Stronger self-control, in turn, is associated with more effective suppression of irrelevant cognitive interference and sustained attentional focus (Andrews-Hanna et al., 2014), which is related to lower levels of mind-wandering. Taken together, these findings suggest that internet addiction is related to cognitive functioning not only directly but also indirectly through a sequential behavioral–cognitive pathway involving physical activity and self-control. This result highlights a dual behavioral and cognitive mechanism underlying the association between internet addiction and mind-wandering and provides a new perspective for understanding cognitive functioning among college students.

### Limitations and practical implications

Although the present study provides insight into the mechanisms underlying the relationship between internet addiction and mind-wandering among college students, several limitations should be acknowledged. First, all variables were assessed using self-report questionnaires, which may be subject to social desirability bias, particularly for constructs such as internet addiction and self-control that carry evaluative connotations. Future studies could incorporate behavioral experiments, experience sampling methods, or physiological indicators to enhance measurement validity. Second, the Cronbach’s α coefficient of the PARS-3 in this study was 0.656, which falls below the conventional threshold of 0.70. Although the composite/index measurement structure of this tool partly accounts for this relatively low value, and previous studies have reported α values close to those in our sample (0.65 and 0.634) (Zhou and Huo, 2022; Chen and Zhang, 2026), this reliability limitation nonetheless warrants caution when interpreting the results, and future studies may supplement self-report measures with objective methods such as accelerometers. Third, the cross-sectional design limits causal inference, and the temporal ordering and long-term effects among variables remain unclear. Although the proposed mediation pathways are consistent with the theoretical model, they cannot be interpreted as evidence of causal ordering or temporal precedence. Therefore, future research adopting longitudinal or experimental designs are therefore needed to more rigorously test the proposed pathway of internet addiction → physical activity → self-control → mind-wandering. Fourth, the sample was primarily composed of college students, which limits the generalizability of the findings to adolescents, older adults, or populations from different cultural contexts. In addition, the grade distribution was highly unbalanced (freshmen accounted for 58.4%, while seniors and postgraduates accounted for only 1.4% and 0.2%, respectively), which may affect the representativeness of the results within the broader college student population. Moreover, online convenience sampling may introduce selection bias—students with higher online activity levels may have been more likely to participate, which is particularly relevant in the context of this study’s focus on internet addiction. Future research should extend the sample to more diverse populations and adopt probability sampling methods (e.g., stratified random sampling) to enhance external validity. Finally, although this study focused on physical activity and self-control, other relevant factors such as sleep quality, emotion regulation, and psychological resilience may also play important roles in the relationship between internet addiction and mind-wandering. Future studies are encouraged to incorporate additional variables to develop a more comprehensive explanatory framework.

Despite these limitations, the study offers both theoretical and practical contributions. At the theoretical level, first, the time displacement hypothesis has typically been used to explain how internet use displaces physical activity time; this study extends this framework further by revealing that this displacement process has downstream cognitive consequences—namely, reduced physical activity not only represents a behavioral change but also depletes self-control resources, ultimately manifesting as increased mind-wandering. Second, the strength model of self-control has traditionally focused on self-regulatory failures in the behavioral domain (e.g., impulse control, addictive behaviors); this study extends its applicability to the cognitive domain, demonstrating that depletion of self-control resources also impairs attentional regulation and facilitates task-irrelevant thinking. Third, and more importantly, this study integrates the above two theoretical perspectives into a chain pathway (internet addiction → reduced physical activity → impaired self-control → mind-wandering), revealing the “behavior–cognition” interaction process underlying the relationship between internet addiction and cognitive functioning. This integrated model transcends previous research paradigms that have examined pairwise relationships in isolation, offering a more comprehensive perspective for understanding the multilevel mechanisms through which internet addiction affects attentional impairment.

From a practical perspective, the findings suggest that simply restricting internet use may not be sufficient to effectively address mind-wandering. Instead, greater attention should be paid to cultivating healthy behavioral habits and self-regulatory capacities among college students. On the one hand, universities could enhance students’ participation in physical activity by improving physical education curricula, enriching campus sports programs, and fostering a supportive exercise environment. On the other hand, self-control training could be integrated into mental health education to help students develop skills in goal setting, time management, and impulse regulation. By jointly promoting physical activity and self-control, it may be possible to more effectively address the cognitive correlates associated with internet addiction.

## Conclusion

The present study examined the relationship between internet addiction and mind-wandering among Chinese college students and elucidated its underlying mechanisms. By constructing a chain mediation model, the findings demonstrated that internet addiction directly predicted mind-wandering and also showed indirect predictive effects through the independent mediating roles of physical activity and self-control. In addition, a significant sequential mediation effect via physical activity and self-control was identified. These findings extend the applicability of the time displacement hypothesis and the self-control strength model, and provide potential pathway-based implications for interventions aimed at addressing cognitive functioning among college students. However, given the relatively modest magnitude of the indirect effects involving physical activity, its practical significance as an intervention target should be interpreted with caution. Given the cross-sectional design, these findings should be interpreted as statistical associations rather than causal evidence. Future research could employ longitudinal or experimental designs to further test and validate the mediating pathways identified in this study.

## Data Availability

The original contributions presented in this study are included in this article/supplementary material, further inquiries can be directed to the corresponding author.
